# CO Gas Sensing Properties of Pure and Cu-Incorporated SnO_2_ Nanoparticles: A Study of Cu-Induced Modifications

**DOI:** 10.3390/s16081283

**Published:** 2016-08-15

**Authors:** Tangirala Venkata Krishna Karthik, María de la Luz Olvera, Arturo Maldonado, Heberto Gómez Pozos

**Affiliations:** 1 Departamento de Ingeniería Eléctrica-SEES, Centro de Investigación y de Estudios Avanzados del Instituto Politécnico Nacional, CINVESTAV-IPN, 14740 Apartado, Mexico; molvera@cinvestav.mx (M.d.l.L.O.); amaldo@cinvestav.mx (A.M.); 2 Área académica de Computación y Electrónica, ICBI, Universidad Autónoma del Estado de Hidalgo, Mineral de la Reforma, 56092 Hidalgo, Mexico; gpozos@uaeh.edu.mx

**Keywords:** gas sensing, tin oxide, CO, copper, doping

## Abstract

Pure and copper (Cu)-incorporated tin oxide (SnO_2_) pellet gas sensors with characteristics provoking gas sensitivity were fabricated and used for measuring carbon monoxide (CO) atmospheres. Non-spherical pure SnO_2_ nano-structures were prepared by using urea as the precipitation agent. The resultant SnO_2_ powders were ball milled and incorporated with a transition metal, Cu, via chemical synthesis method. The incorporation is confirmed by high-resolution transmission electron microscope (HRTEM) analysis. By utilizing Cu-incorporated SnO_2_ pellets an increase in the CO sensitivity by an order of three, and a decrease in the response and recovery times by an order of two, were obtained. This improvement in the sensitivity is due to two factors that arise due to Cu incorporation: necks between the microparticles and stacking faults in the grains. These two factors increased the conductivity and oxygen adsorption, respectively, at the pellets’ surface of SnO_2_ which, in turn, raised the CO sensitivity.

## 1. Introduction

Gas leak detection is a constructive way of testing dangerous toxic gases from sealed elements. A common industrial hazard gas produced from many systems starting from the burning of a cigarette to gasoline is carbon monoxide (CO) [1].

In order to examine the gas response of the oxidizing and reducing gases, metal oxides are very generally utilized mainly due to the available oxygen vacancies at the surface [2]. The basic principle of a gas sensor is that the atmospheric oxygen adsorbs on the metal oxide surface at the elevated temperatures. Later, the reaction of gas molecules with the pre-adsorbed oxygen results in the change in the conductivity of the surface of the metal oxide [3]. The first considered metal oxide for gas sensor applications was tin oxide (SnO_2_) and is the material frequently used until now due to both its dual valance and the adjustable surface oxygen concentration [4].

Pellets consisting of SnO_2_ powders are more practicable for gas sensors than thin films due to their higher porosity, surface area, and no substrate effects. Various methods have been employed to date for preparing tin oxide powders, like microwave synthesis [5] and sol-gel methods [6], etc. Homogenous precipitation, employing urea as the precipitant agent is widely known and illustrious in synthesizing novel phases and fine particulate materials. Hydrolysis of urea requires a moderate temperature process (80–100 °C) which grants coarse powders with adequate characteristics to be used in gas sensing applications [7,8].

One way to increase the sensor´s sensitivity is by decreasing the particle size, which is very hard to control in practice. The other way is to modify or control the surface properties of the material, which is generally performed by doping or by incorporating with metals in the base material. A frequently used method for adding catalysts is during the synthesis of the metal oxide semiconductor (MOS), which is known as chemical doping. In this method, the catalysts are considered to be located in the substitutional or interstitial positions of the semiconductor. The stress and strain produced due to the structural changes will increase the oxygen adsorption which, in turn, increases the gas sensitivity [9,10]. Cu is the most used transition metal for incorporation in tin (Sn) because of its comparable radius, as the ionic radius of Sn^4+^ and Cu^2+^ are around 0.071 and 0.072 nm, respectively [11,12]. Therefore, in our case, SnO_2_ acts as a gas-sensing matrix and Cu will act as a structure modifier increasing the surface reactivity with the gases. However, a systematic study of the influence of catalysts on the gas-sensing properties is still missing.

This paper will give a systematic and detailed study about the effect of catalyst and incorporation methods on the gas-sensing properties of SnO_2_ pellets. Primarily, we explain the structural properties of the pure and Cu-incorporated pellets. Later, the changes observed due to the Cu incorporation in the crystal structure, and also on the particles’ surface, were revealed by scanning electron microscopy (SEM) and HRTEM analysis. Subsequently, the most important properties of a gas sensor, such as sensitivity, response, and recovery times, were also reported. Finally, a well-substantiated explanation for achieving the highest sensitivities is given by comparing the structural, morphological, and sensing properties with the established sensing mechanism.

## 2. Experimental Procedure

### 2.1. Homogeneous Precipitation of Tin Oxide Powders

Primarily, aqueous solutions of tin chloride pentahydrate, SnCl_4_∙5H_2_O (J. T. Baker, Center Valley, PA, USA), and urea, CH_4_N_2_O, (Sigma Aldrich, St. Louis, MO, USA, Calle 6 Norte 107, 50200 Toluca de Lerdo, Mexico) with 0.4 molar concentration were prepared. Later, 1:2 mixtures from solutions with equal molar concentrations (volume proportion: 30 mL of tin chloride solution and 60 mL of urea solution) was prepared. Subsequently, the mixed solution was stirred and heated until a temperature of 93 ± 5 °C was reached and maintained until the precipitate was formed. This increase in temperature is for the decomposition of urea and formation of precipitate. The resultant precipitates were washed several times by using a ROTINA-420R centrifuge in order to remove all the residues, especially chlorine. The precipitates were centrifuged at 400 rpm for 1 h until the pH of the supernatant reached 12. The resultant pastes were dried in air at 100 °C for 24 h in order to eliminate the aqueous solvents. Finally, all powders were calcined in a furnace at 800 °C for 2 h in a normal atmosphere, during which SnO_2_ particles were formed.

### 2.2. Cu-Incorporated SnO_2_ (Cu:SnO_2_) Powders

Aqueous solution of tin chloride pentahydrate, SnCl_4_∙5H_2_O (J. T. Baker), with 0.4 mol concentration was prepared. 1 wt % copper chloride, CuCl_2_ (Sigma Aldrich) powders was added to the previously prepared tin chloride solution. Later, to obtain the Cu-incorporated SnO_2_ powders by urea, the above feedstock solution was treated in the same way as explained in the case of pure powders. The copper incorporation, either in matrix or in surface-like clusters, occurred during the calcination at 800 °C of the dried powders. All of the incorporated powders obtained were ball milled for 6 h at 400 rpm. Finally, all of the obtained powders were subsequently pressed using a manual pressing machine with a pressure of 16 tons for 90 min.

## 3. Characterization

X-ray diffraction (XRD) analysis using a PANalytical diffractometer with CuKα at 20 mA and 40 KV was carried out to identify the phase compound and the crystalline structure of the SnO_2_ powders. The morphological characteristics and the particle size of the calcined particles were examined with scanning electron microscopy (SEM), by using AURIGA equipment. All Cu-incorporated SnO_2_ powders were analyzed with a high-resolution transmission electron microscope (HRTEM JEM-ARF 200F) to confirm the crystal size, identify the catalyst’s locations, and also the lattice spacing between the SnO_2_ crystal planes.

**Figure 1:**
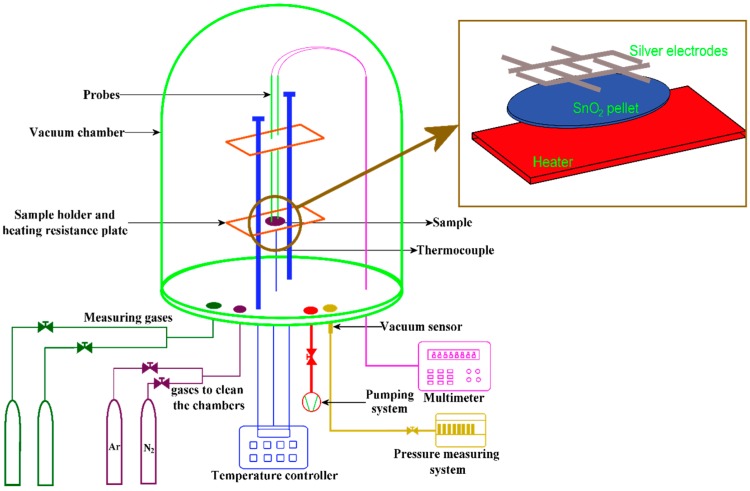
Gas sensing system.

For measuring the gas sensing response in CO, pure silver ohmic contacts, by using a pellet holder and a steel mask, were deposited on the pellets’ surface by the thermal evaporation technique. The schematic diagram of the pellet gas sensor is shown in Figure 1.

The measurements were made at three different operating temperatures: 100, 200 and 300 °C. The conductance changes were registered by using a Keithley 2001 multimeter. For controlling the partial pressure in the chamber a TM20 Leybold detector was used. Once the sample is placed, the chamber pressure is brought to 70 Pa and different amounts of 99.5% pure CO is passed to the chamber. The change in pressure imparts the concentration of CO. The sensitivity of the pellets, “S”, was obtained by calculating the electrical conductance ratio in air (3066.414 Pa), G_O_, and in the presence of different concentrations of CO, 1, 5, 50, 100, 200 and 300 ppm, G_G_.

(1) $$S=\frac{G_{G}-G_{O}}{G_{O}}$$

## 4. Results and Discussion

### 4.1. X-ray Diffraction Analysis

**Figure 2:**
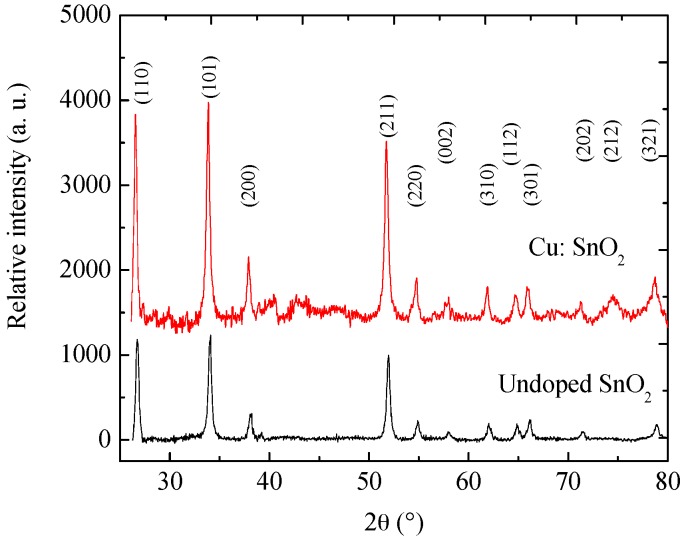
XRD patterns of pure and Cu-incorporated SnO_2_ powders.

In this work, pure and Cu-incorporated SnO_2_ nano-crystals were synthesized. Figure 2 depicts the XRD patterns obtained for the pure and Cu-incorporated SnO_2_ powders. For both pure and incorporated SnO_2_ powders, the signal associated with (110), (101), and (211) planes prevailed over the rest of the encountered reflections, although slightly higher in the (200), (220), (002), (310), (112), (301), (202), (212) and (321) signals that were observed for the Cu-incorporated SnO_2_ films. However, the peak intensities represent the quantity of powders during the measurement. Additionally, it is evident that all of these peaks exhibit the pure tetragonal rutile phase of SnO_2_, according to JCPDS Card 077-0450 [13]. All pure and incorporated calcined powders synthesized showed a well-defined crystallinity, and no other peaks, like Sn, SnO, Sn_2_O_3_, Sn_3_O_4_, or products related to Cl (which confirms the removal of Cl during the centrifugation) can be identified; therefore, mono-phase, pure SnO_2_ powders are synthesized. Additionally, in the case of Cu-incorporated SnO_2_ powders, no other Cu or oxides of Cu peaks were observed in the patterns. This may be due to the utilization of a low amount of metallic ions during incorporation (1 wt %).

From Figure 2 it is observed that the XRD peak intensities of the incorporated powders show an upper shift in the peak positions compared to those of pure powders. In general, the peak intensity depends on two factors: one is the quantity of powder utilized during the analysis and another is lattice structure discrepancies [14]. Since Cu has a comparable ionic radius as Sn, the replacement of Sn with Cu and the ball milling process resulting in a compressive strain in the lattice, which would have caused an upper shift in the XRD peaks for the Cu-incorporated SnO_2_ powders. In order to ascertain the effect of Cu on the structural characteristics, crystallite size (D), lattice constants (a and c), crystal volume (V), porosity (P) [15], and texture coefficient (T_C_(hkl)) were calculated by using Equations (2)–(7) and reported in Table 1:

(2) $$D=\frac{0.89\lambda }{\beta \text{Cos}\theta }$$

(3) $$V=a^{2}c$$

(4) $$P=\left(1-\frac{\rho_{a}}{\rho_{x}}\right)\times 100\%$$

(5) $$\rho_{a}=\frac{m}{v}$$

(6) $$\rho_{x}=\frac{\text{nM}}{\text{NV}}$$

whereas λ is the wavelength of incident X-ray (λ = 0.15418 nm), β is full width at half maximum (FWHM) intensity, θ is the Bragg’s diffraction angle in radians, a and c are lattice parameters, m and v are the mass and volume of the samples, n is the number of molecules per unit cell, M is the molecular weight, and N is Avogadro’s number.

The preferred orientation of SnO_2_ crystal planes can be quantitatively evaluated using the texture coefficient, T_C_ (hkl), which has been determined from expression [16], shown in Equation (7), whereas I (hkl) is the relative intensity of a plane (hkl), and I_O_ (hkl) is the standard intensity of the plane (hkl) taken from the JCPDS data [13]. T_C_ (hkl) = 1 implies that the sample presents random crystallites; whereas T_C_ values lower than 1 means that there are an lack of grains oriented with that (hkl) direction.

(7) $$T_{C}\left(\text{hkl}\right)=\frac{\frac{I\left(\text{hkl}\right)}{I_{o}\left(\text{hkl}\right)}}{\left(\frac{1}{n}\right)\sum_{n}\frac{I\left(\text{hkl}\right)}{I_{o}\left(\text{hkl}\right)}}$$

By comparing Table 1 and Figure 2, we observe a minute increment in the lattice parameters and crystallite size of Cu-incorporated SnO_2_ powders compared to the pure powders. This can explain the lack of any peak shifts in the XRD patterns. This increase is due to the increase in the ionic radii of the catalyst. In case of Cu, as it replaces a Sn atom due to it its higher ionic radius, the crystal distance increases which, in turn, increases the lattice parameters of the unit cell, crystallite size and, further, the volume. An increase in the volume theoretically decreases the density of the particles on the surface, which results in a porous surface. From Table 1 we can observe that the porosity value was increased around 40% for Cu-incorporated SnO_2_. In general, with the increase in the porosity the sensitivity of a gas sensor also increases.

Additionally, T_C_ values calculated for the maximum peak emission, which correspond to the orientation (101) of SnO_2_, Table 1, it is observed that the values of T_C_ (101) for both Cu-incorporated and undoped SnO_2_ are less than 1, which means that the orientation (101) is not preferential and Cu-incorporated SnO_2_ powders show lesser T_C_ (see Table 1) compared to pure SnO_2_ powders, which confirms that Cu incorporation worsens the crystallinity. In addition to porosity, particle size, surface morphology, and measuring temperature sensitivity also depend on crystallinity.

### 4.2. SEM Analysis

**Figure 3:**
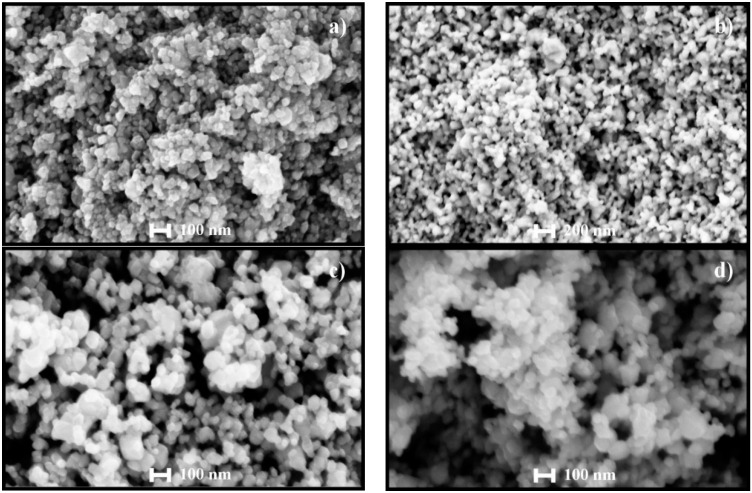
SEM images of pure SnO_2_ powders (**a**) before and (**b**) after ball milling; and Cu-incorporated SnO_2_ powders (**c**) before and (**d**) after ball milling, respectively.

The surface morphology of the pure and Cu-incorporated SnO_2_ powders, with respect to the effect of ball milling, is shown in Figure 3. Figure 3a,b correspond to pure SnO_2_ powders, before and after ball milling, respectively. From these micrographs we can observe that, the powders before ball milling consists of agglomerated small particles, most of which are smaller than 50 nm. However, the powders after the ball milling consist of particles with sizes around 100 nm, with the exception of some particles as small as 50 nm in diameter. This increase in the particle size is due to the solid state reactions of SnO_2_ species, promoted by the heat produced in the jar during the high energy grinding process. We also observe that, for the ball-milled powders, all particles were connected to each other through neck formation. During the ball milling process, the heat produced in the jar acts as an additional heat treatment process. As the particle size reduces during the milling process, the smaller particles have higher driving forces to transfer the atoms in the solid state, which results in the formation of the necks between the particles [17]. According to Weaver et al. [17], the surface atoms of a particle gain the kinetic energy with the additional heat treatment, therefore, particles primarily adhere to each other and finally result in the formation of the necks. The neck formation is a good sign for the gas sensitivity, because the necks act as channels between particles to increase the conductivity of the pellet.

Figure 3c,d reveal the morphology of the Cu-incorporated SnO_2_ powders. Primarily, the particle size has increased slightly compared to the pure powders, in both milled and un-milled powders. This is in good agreement with the theoretically-calculated crystallite sizes of the pure and incorporated powders from the XRD data (refer to Table 1). The particle size obtained was around 30–80 nm for the incorporated powders. We can also observe that there is neither particle size change nor the neck formation, like in pure powders, after the ball milling. Since we have employed chemical doping methods to incorporate Cu, in which the additives are added during the synthesis process, they can be incorporated into the lattice or reside on the surface of metal oxide [10]. An inhibition of the coalescence for Cu-incorporated powders is observed. The catalysts reside on the surface and further inhibit the particle size and further coalescence [18].

Another important detail observed from Figure 3 is that the incorporated powders are more porous compared to the pure powders. Due to incorporation or presence of catalysts on the surface, the particle size and porosity are increased. More porous structures facilitate the gas to interact with 2–3 monolayers of the pellet, which results in higher sensitivity of the pellets. In order to have detailed structural analysis of the Cu incorporation, HRTEM analysis was performed.

### 4.3. EDAX Analysis

**Figure 4:**
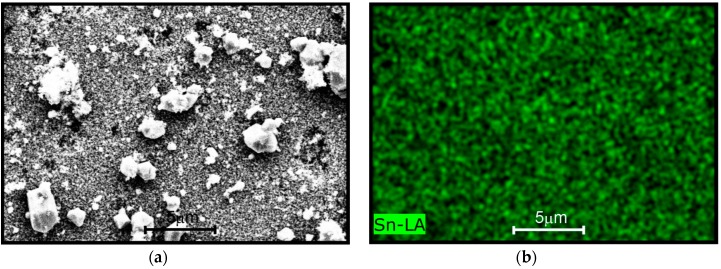

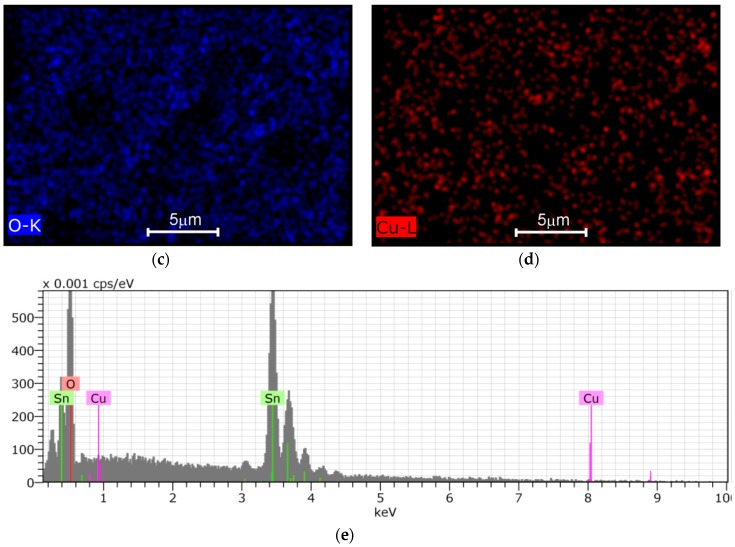
EDAX analysis of (**a**) Cu:SnO_2_ powders surface with clusters (**b**) Sn; (**c**) O; (**d**) CuO; and (**e**) composition plot with inset confirming the wt % of each element.

The presence of Cu, Sn, O from Figure 4 is evident. From Figure 4a–d, it can be observed that, the Sn, O, and Cu were homogenously distributed all over the surface. Figure 4e shows the presence of copper and its corresponding wt % around 0.99. Additionally, from Figure 4e, the Sn/O wt % ratio is approximately ½, which affirms the SnO_2_ distribution. From SEM and EDAX analysis, we can conclude that homogeneous powders with nano-particles were successfully synthesized by the cost-effective technique.

### 4.4. XPS Analysis

**Figure 5:**
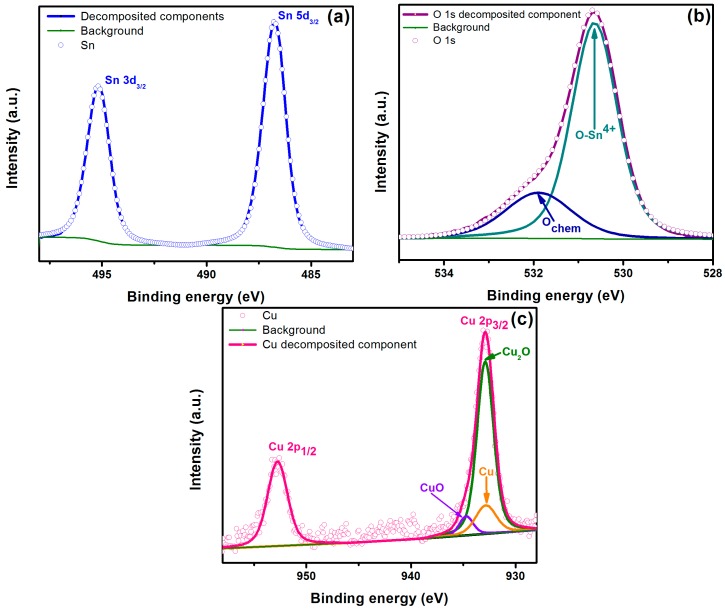
XPS spectra of Cu-doped SnO2 pellet surface: (**a**) Sn 3d; (**b**) O 1s; and (**c**) Cu 2p.

To gain more insight into the chemical composition of the dopant in Cu:SnO_2_ powders, we also carried out XPS measurements on the fabricated Cu:SnO_2_ pellets. The two strong peaks at around 486.5 and 495 eV displayed in Figure 5a can be attributed to Sn 3d3/2 and 3d5/2, respectively [19]. In Figure 5b the XPS O1s line of a Cu:SnO_2_ pellet is presented. In this case, the primary spectrum of the XPS O1s peak shows that it is wide, asymmetrical, and exhibits an evident shoulder at the high binding energy side of the spectrum, which confirms the components corresponding adsorbed oxygen or hydroxide (OH) components resulted due to the precursors utilized [20]. The Cu 2p peak in the XPS spectrum is shown in Figure 5c; the two peaks centered at 933.5 and 953.2 eV were assigned to Cu 2p3/2 and Cu 2p1/2 of Cu_2_O, respectively, and the three smaller peaks, obtained by deconvolution of the major peak Cu 2p3/2, appearing at 932.7, 932.8, and 952.2 eV correspond to Cu_2_O, Cu, and CuO [21]. The curve fit results of the Cu 2p core level XPS measured on the surface of the pellet reveal that the predominant phase was Cu_2_O, constituting about 80% on the surface. During the sensing measurements, at higher temperatures Cu_2_O changes to Cu or CuO when reacted with CO or O (refer Equations (8) and (9), respectively). During the decrease in the temperature, CuO returns back to the Cu_2_O phase due to the oxygen desorption (refer Equation (10)), whereas the Cu remains metallic, which is also confirmed by XPS (refer Figure 5c) [22].

Cu_2_O + CO = 2 Cu + CO_2_ (8)

Cu_2_O + O = 2 CuO (9)

2 CuO − 1 O = Cu_2_O (10)

### 4.5. HRTEM Analysis

**Figure 6:**
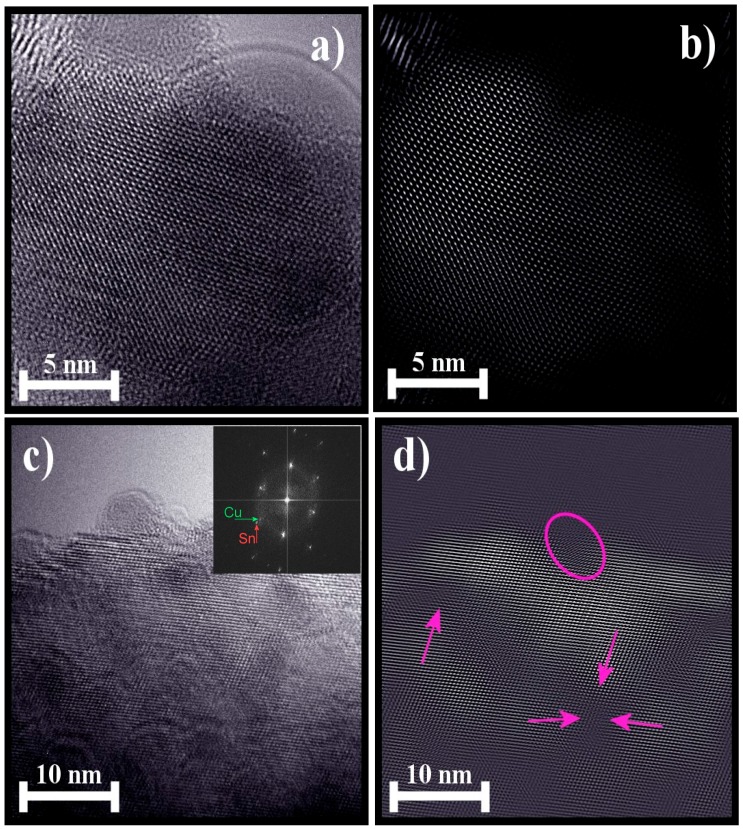
(**a**) HRTEM image of pure SnO_2_ powders; inset: the corresponding SAED pattern; (**b**) the reconstructed image of (**a**); (**c**) HRTEM images of Cu-incorporated SnO_2_ powders; inset: the corresponding SAED pattern; and (**d**) the reconstructed HRTEM of (**c**).

Figure 6a,c show the pure and Cu-incorporated SnO_2_ particle surfaces, respectively. Their corresponding reconstructed images are show in Figure 6b,d. The insets in Figure 6a,c are the corresponding selected-area electron diffraction (SAED) patterns. Figure 6a shows that all of the atomic planes were well defined without any discrepancies and correspond to SnO_2_ crystal planes. The d-spacings measured from the reconstructed image (Figure 6b) are in good agreement with those of the (110) plane of cassiterite SnO_2_ (JCPDS Card 077-0450 card) [13], corresponding to the tetragonal rutile crystal structure (space group = P42/mnm). From Figure 6c, we can observe that various smaller particles around 5 nm were beneath the focused particle.

In order to observe more clearly the effect of the catalyst, the image is reconstructed by masking and shown in Figure 6d. Localization of Cu on the surface (pink circle) and various stacking faults, like surface undulations (pink arrows), were highlighted in the reconstructed image (Figure 6d). It is confirmed that some amount of catalyst (Cu) is localized on the surface of the SnO_2_ particle. By considering the amount of catalyst (1 wt %) utilized, which is very low, a few identities of catalyst were observed. The inter-plane distance measured for the copper particle corresponds to the (111) plane of cubic Cu (JCPDS Card 00-004-0836) [23].

Additionally, primarily, the (110) inter-plane distance is estimated to be 0.335 and 0.337 nm for the pure and Cu-incorporated powders, respectively. This increase in the lattice parameter suggests an increase of the unit cell volume for the incorporated powder. Secondarily, we can observe the copper undulations on the SnO_2_ crystal, which are highlighted with pink arrows. From these two factors, it can be considered that the Cu insertion in the host (SnO_2_) matrix is possible.

The precise position of copper’s presence is shown in the SEAD patterns (Figure 6c inset). The estimated lattice spacing values from the HRTEM are in consistent with the calculated XRD data. Therefore, it can be stated from the HRTEM and XRD analysis that a part of the catalysts utilized were induced into the SnO_2_ matrix and have created stacking faults like undulations in the SnO_2_ lattice, whereas small Cu clusters of catalyst, residing on the surface of the SnO_2_ grains were also formed. SnO_2_ crystals with a Sn excess can show defects as stacking faults. Electronic effects were produced due to Sn excess, which enhances the bulk conductivity and, subsequently, gas sensitivity [24]. Additionally, the metal clusters assure the possibility of a spill-over mechanism of gas sensing [25].

### 4.6. Gas Sensing Properties

**Figure 7:**
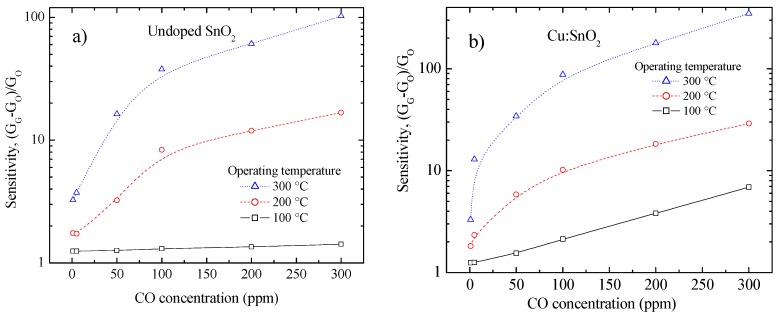
CO sensing response of (**a**) pure and (**b**) Cu-incorporated SnO_2_ pellets.

In order to characterize the performance of the sensors composed of pure and Cu-incorporated SnO_2_ pellets, the sensors were exposed to a range of CO gas concentrations (0–300 ppm) at various temperatures (100, 200 and 300 °C). Figure 7a,b show the typical sensor responses of pure and Cu-incorporated SnO_2_ composites. The sensing behavior of both sensors was similar to that of a typical n-type oxide semiconductor (i.e., a decrease in electrical resistance by a reducing gas). The responses of both sensors monotonically increased with increasing CO gas concentration and temperature. Reaction of carbon monoxide molecules with adsorbed oxygen can be expressed in Equation (8) [26]:

(11) $$2\text{CO}+O_{2}^{-}{}_{\left(\text{ads}\right)}\to /\text{Heat}/\to 2{\text{CO}}_{2}+e^{-}$$

As the gas concentration increases, the number of molecules reacting with the surface increases, which increases the sensitivity [27]. When the operating temperature of the gas sensor increases, the oxygen adsorbed is more reactive (O^−^ or O^2−^) with the reducing gas, which increases the sensitivity [28].

Notably, Cu-incorporated pellets exhibited greater sensing responses than the pure SnO_2_ at all temperatures, even to very small amounts of CO (1 ppm). The maximum gas sensing response of pure and Cu-incorporated SnO_2_ pellets were 102.5 and 348.4, respectively, to 300 ppm of CO gas at 300 °C (Figure 7a,b). More association between the particles (refer Figure 3) and the surface undulations (refer Figure 6) have improved the surface conductivity and oxygen adsorption respectively of the incorporated pellets, which successively ensued in higher sensitivities than the pure pellets. Low temperature (at 100 °C) sensitivities were also obtained (refer to Figure 7) for Cu-incorporated SnO_2_ pellets, and such composite devices can be used as cost effective, VLSI-integrable CO_2_ sensors, operational at relatively low temperatures.

### 4.7. Response and Recovery Times

The time required for attaining 90% of the final electrical resistance with the test gas is referred to the sensor response time (τ_res_), and the time taken to retrieve 10% of the saturation electrical resistance in the absence of the test gas is referred to sensor recovery time (τ_rec_). In addition to having higher sensitivities, it is significant to have shorter response and recovery times for an acceptable sensor.

**Figure 8:**
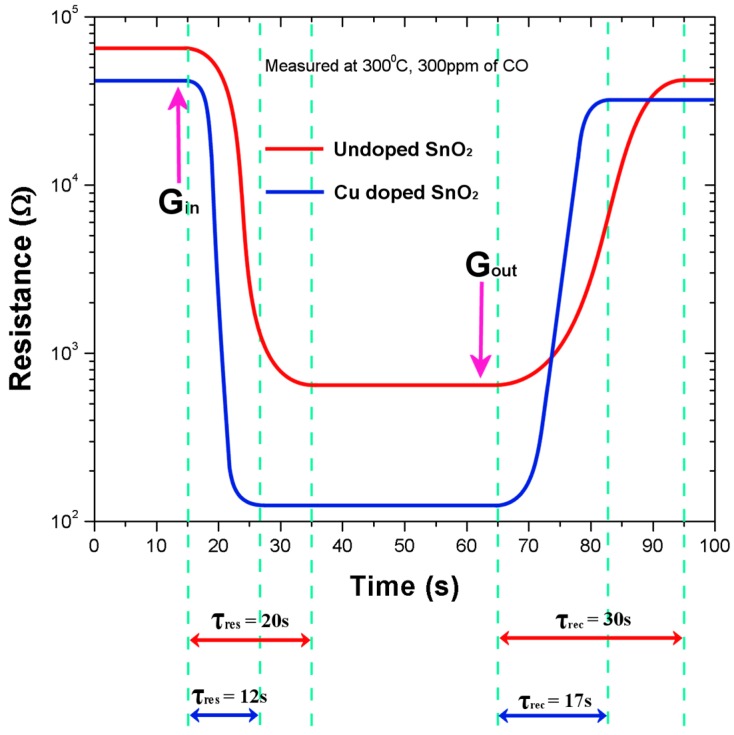
The response (τ_res_) and recovery (τ_rec_) times of the pure and Cu-incorporated SnO_2_ pellets.

**Figure 9:**
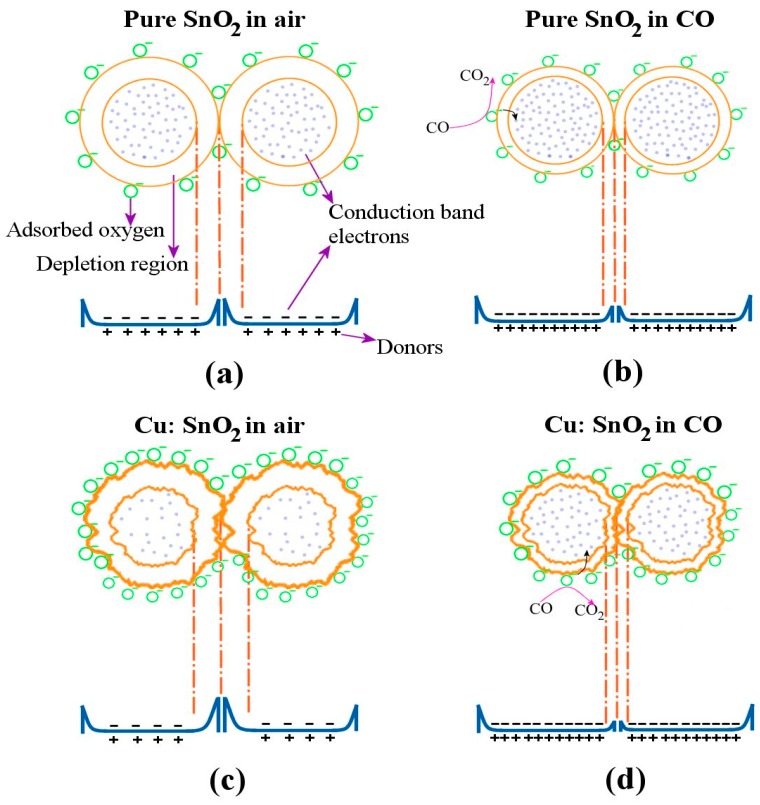
Gas-sensing mechanism of the pure SnO_2_ in (**a**) air and (**b**) CO; and Cu:SnO_2_ pellets in (**c**) air and (**d**) CO.

Figure 8 below shows the response and recovery times of the pure and copper-incorporated SnO_2_ pellets measured at 300 °C and for 300 ppm of CO gas concentration. The response and recovery times of Cu:SnO_2_ have reduced by an order of 2. Response and recovery times with their corresponding sensitivities are summarized in Table 2. The reason for the increase in the sensitivity and decrease in the response and recovery times is due to the structural and morphological changes occurring in the Cu-incorporated SnO_2_ powders. Figure 9 illustrates the gas-sensing concept in Cu:SnO_2_ and pure SnO_2_ powders.

Figure 9a,b shows the pure SnO_2_ grains’ reaction with the air and CO, respectively. The depletion region decreases (Figure 9b) as the CO reacts with the adsorbed oxygen, which results in the decrease in the barrier height and a further increase in sensitivity in the presence of gas. Whereas, for the pellets manufactured with Cu:SnO_2_, the surface of the grains is rougher (Figure 9c) due to the undulations resulting from the copper incorporation (Figure 6). This increased surface roughness increases the oxygen adsorption and further CO sensitivity. The decrement in the barrier height observed in the presence of the gas is higher than the former case. This, besides increasing sensitivity, accelerates the adsorption and desorption surface reactions, such as the rate of chemical reaction between CO molecules and chemisorbed O^−^. Furthermore, copper does not change its free energy in chemical reactions, but decreases the activation energy which, subsequently, increases the sensor’s response time [29].

## 5. Conclusions

Pure and Cu:SnO_2_ powders were successfully synthesized using homogeneous precipitation using urea as the precipitation agent. The CO-sensing properties of pure and Cu-incorporated SnO_2_ pellets, with respect to the operating temperature and gas concentration, were analyzed. Pure SnO_2_ and Cu:SnO_2_ pellets show similar XRD patterns, but the crystal structure of the pure SnO_2_ pellets is modified slightly with the introduction of the Cu, lattice parameters, and the porosity increased.

Pure and Cu:SnO_2_ pellets exhibit sensitivity values around ~100 and 350, respectively, at 300 °C, for a CO concentration around 300 ppm. This increase is due to the Cu incorporation modifying the surface morphology of the grains in different ways, the formation of the necks (SEM analysis) and undulations produced on the surface (HRTEM analysis), and also the incorporated powders are more porous compared to the pure powders, which is also a good sign for the gas sensitivity.

From the HRTEM measurements, it is observed that Cu incorporation in SnO_2_ created stacking faults like undulations in the SnO_2_, with Sn-enriched stoichiometry. Electronic effects were produced due to Sn enrichment, which enhances the bulk conductivity and, subsequently, gas sensitivity. The presence of copper in the SnO_2_ favored in decrementing the barrier height, which increased the conductivity and further sensitivity. Additionally, the copper-incorporation-produced undulations accelerated the oxygen adsorption and, subsequently, decreased the response and recovery times of the sensor.

## Figures and Tables

**Table 1:** Crystallite size (D), lattice constants (a and c), volume of the crystal (V), porosity (P), and texture coefficient (T_C_ (101)) of the pure and Cu-incorporated SnO_2_ nano-crystals.

SnO_2_ Type	a (nm)	c (nm)	D (nm)	V (10^−24^ cm^3^)	P (%)	T_C_ (101)
Pure	0.4749	0.3185	26.27	71.40	32.30	0.921
Cu incorporated	0.4756	0.3192	26.32	71.66	58.35	0.136

**Table 2:** Sensitivity, response, and recovery times of pure SnO_2_ and Cu:SnO_2_ pellets.

SnO_2_ Pellet	Maximum Sensitivity	Response Time (s)	Recovery Time (s)
Pure SnO_2_	102.8	18	27
Cu:SnO_2_	348.4	10.8	15.3
